# Editorial: Reproductive Barriers and Gene Introgression in Rice Species

**DOI:** 10.3389/fpls.2021.699761

**Published:** 2021-08-11

**Authors:** Dayun Tao, Kenneth L. McNally, Yohei Koide, Kazuki Matsubara

**Affiliations:** ^1^Yunnan Key Laboratory for Rice Genetic Improvement, Food Crops Research Institute, Yunnan Academy of Agricultural Sciences, Kunming, China; ^2^T. T. Chang Genetic Resources Center, International Rice Research Institute, Los Baños, Philippines; ^3^Laboratory of Plant Breeding, Research Faculty of Agriculture, Hokkaido University, Sapporo, Japan; ^4^Institute of Crop Science, National Agriculture and Food Research Organization, Tsukuba, Japan

**Keywords:** rice, reproductive barriers, wide hybridization, gene introgression, wild species

Rice (*Oryza sativa* L.) is one of the most widely consumed cereals both in developing and developed countries with the third-highest worldwide production (FAOSTAT, http://www.fao.org/faostat/en/#data). Rice production is constantly subject to multiple environmental stresses, both biotic and abiotic. In addition, the effect of such stresses changes in response to environmental conditions. The increasing global food demand, together with rapid population growth forces rice geneticists and breeders to speed up and push forward the improvement of resistance and/or avoidance to such stresses as well as productivity (International Rice Research Institute, https://www.irri.org/world-food-day-2020).

To achieve this complex task, the use of distant relatives of rice as donor of genetic diversity has been attempted to improve the resistance together with productivity in rice varieties, because of the limited genetic variation within current rice varieties (Brar and Khush, 1997, 2018). However, the introgression of useful genes from distant relatives of rice is often prevented by interspecific or intersubspecific reproductive barriers (Oka, 1988).

Various types of reproductive barriers can occur at different stages during the life cycle (Rieseberg and Willis, 2007). Depending on the developmental stage, reproductive isolation can be categorized as prezygotic and postzygotic reproductive isolation (Smith, 1989). Prezygotic reproductive isolation prevents the formation of hybrid zygotes, while postzygotic reproductive isolation results in hybrid incompatibility, including hybrid necrosis, weakness, sterility, and breakdown (Stebbins, 1950). Postzygotic reproductive barriers are the major obstacles for transferring favorable genes from wild and the cultivated relative to cultivated rice. Understanding the causes and consequences of reproductive isolation, and providing a rational breeding strategy, may improve cultivated rice for various traits of importance to rice improvement, such as yield, quality, biotic and abiotic resistance.

In the last few decades, much progress has been made in these research areas, especially in mapping genes associated with reproductive barriers and resistance to environmental stresses (Brar and Khush, 1997, 2018; Ouyang and Zhang, 2018). The Research Topic entitled “Reproductive Barriers and Gene Introgression in Rice Species” is meant to gather together current knowledge in this field and share it with the scientific community to accelerate rice genetic improvement.

In this Research Topic, Sakata et al. isolated two novel loci *S22A* and *S22B* for hybrid pollen sterility between *O. sativa* and *O. glumaepatula*. Both genes encoded proteins containing DUF1668 domains that were expressed gametophytically in anthers, which is Poaceae species specific DUF. Functional diversifications of duplicated genes in different rice species could lead to hybrid sterility or breakdown as observed in cases of *S27*/*S28, DPL1*/*DPL2*, and *DGS1*/*DGS2* loci (Mizuta et al., 2010; Yamagata et al., 2010; Nguyen et al., 2017). The only common feature among these hybrid incompatibility systems is that they are caused by gene duplication/reciprocal loss-of-function mutations, but their encoding proteins are different. In *S22A* and *S22B*, the duplicated DUF1668-domain genes may have provided genetic potential to induce hybrid incompatibility by consequent mutations after the wild species' divergence. Unraveling the molecular mechanism of the *S22* locus will help us understand the nature of hybrid sterility.

Hybrid sterility is the major bottleneck for introgressing favorable genes from wild relatives of rice into modern varieties (see Li et al. for a review). Many scientists have devoted their careers in developing strategies to overcome hybrid sterility in rice such as the bridging-parent strategy, using wide-compatibility genes, and gene-editing approaches. Kuniyoshi et al. discovered a new approach for enhancing the hybrid fertility via pollen culture (see also Koide et al.). Abnormal meiosis in the first division restitution (FDR) and/or the second division restitution (SDR) resulted in various ploidy levels of the regenerated plants. Early rescue of microspores carrying diploid gametes led to increased fertility. This study provided a new vision to overcome hybrid sterility.

Furthermore, researchers contributed various kinds of knowledge about other reproductive barriers and reproductive biology: transmission ratio distortion (Zhang et al.), hybrid incompatibility (with special emphasis on autoimmunity in hybrids, Calvo-Baltanás et al.), hybrid breakdown (Munguambe et al.; Matsubara), male and female development genes—*OsMFS1*/*OsHOP2* (Lu et al.), and self-incompatibility (Lian et al.) in this Research Topic.

Chromosome segment substitution line libraries (CSSLs) are a powerful genetic resource for systematic genetic dissection of agronomic traits and varieties improvement in rice. Singh et al. produced six interspecific introgression libraries derived from crossing three wild species as the donor parents with two cultivated varieties as the recurrent parents. The three wild AA genome species originated from different regions and represented abundant genetic diversity; the popular *indica* cv. IR 64 and *japonica* cv. Cybonnet constituted the domesticated *O. sativa* backgrounds. This study contributed to understand the effects of wild introgressions in *indica* and *japonica* backgrounds, respectively. The genes for pericarp color, hull color, shattering, flowering time and seed production were confirmed using introgression libraries. Further, it is great news that seeds from the six CSSLs are available from the Genetics Stocks-Oryza (GSOR) center at the Dale Bumpers National Rice Research Center in Stuttgart, AR, United States. Mussurova et al. introduced the concept of platinum standard reference genome sequences—a new standard by which contiguous near-gap free reference genomes of wild relatives. Such genomic resources will much facilitate the introgression of useful genes from wild relatives into cultivated rice.

Molecular marker assisted (MAS) selection promotes faster breeding of rice lines carrying introgressions of rice resistant genes into recipient parents (see Ramalingam et al.). For enhancing resistance to bacterial blight, blast, and sheath blight in rice, a pyramiding scheme was adopted where 1) three genes for bacterial blight, *xa5, xa13*, and *Xa21*, were introgressed into recurrent parents by a first cycle of MAS, and 2) the *Pi54* gene for blast and three QTLs for sheath blight (*qSBR7-1, qSBR11-1*, and *qSBR11-2*) were introduced into the improved lines by the second cycle of MAS. Such rapid-stacking, pyramiding strategies are now widely used to produce commercial and released varieties both in the private sector and, increasingly, in the public sector (e.g., at IRRI and AfricaRice through the Bill and Melinda Gates Foundation and USAID funded projects). Uddin and Fukuta reviewed quantitative trait loci related to differentiation between lowland and upland ecotypes. Such information must be also very useful for improvement of rice adapted in different environments.

The African domesticated species, *O. glaberrima*, possesses many valuable traits for tolerance to biotic and abiotic stresses and comprises an elite gene pool for improving Asian cultivated rice. Dong et al. identified *Pi69*(t), a novel gene for rice blast resistance that was mapped to a 0.9 cM region on the long arm of chromosome 6. Lines carrying *Pi69*(t) were resistant to 148 of 151 blast strains collected from 6 countries, suggesting that *Pi69*(t) confers broad-spectrum resistance to blast and is a valuable gene for blast-resistance breeding. This is the first blast resistance gene identified from *O. glaberrima*. In summary, this Frontier hot issue provides an up-to-date account of some of the exciting areas of current research related to reproductive barriers and introgression in rice, particularly by virtue of the effort of young researchers. Both the research and review publications shed light on the nature of reproductive barriers and provide new insights for rice variety improvement.

Lastly, we dedicate this issue to the memory of Dr. Darshan Singh Brar (March 7, 1944–March 11, 2020) (Figure 1). Not only was Dr. Brar a globally recognized plant breeder and cytogeneticist, but he was also a respected colleague, teacher, mentor, friend. His career at IRRI (1987–2012) centered around using wild species as donors for important traits for rice improvement, understanding reproductive barriers, and rice diversity. He never sought the limelight, preferring to focus diligently on his work. His insight and tutelage inspired many, his humble and kind nature are sorely missed, and his humor made the days glow. Dr. Brar, we will never forget you; our lives were enriched by the time we were fortunate to have had with you.

**Figure 1:**
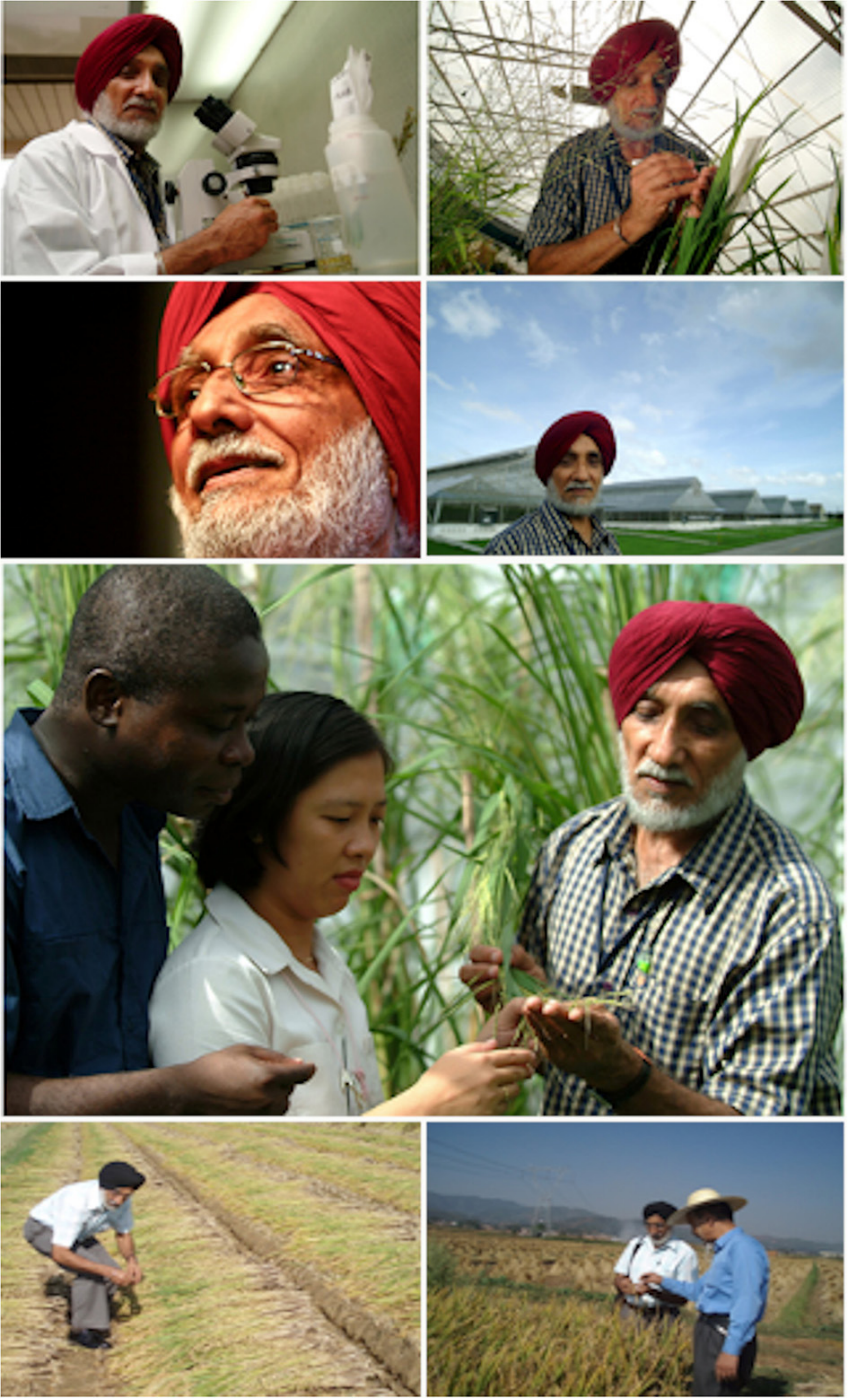
In memory of Dr. Darshan Singh Brar. From top left to bottom right: Dr. Brar (1) the cytologist, (2) the geneticist, (3) the wise, (4) the contemplator, (5) the mentor (with Dr. Kofi Bimpong and Ms. Joie M. Ramos), (6) the breeder, (7) the teacher (with Mr. Jiawu Zhou). Pictures 1–5 are from the IRRI archives (see http://news.irri.org/2020/03/dr-darshan-brar-leading-irri-breeder.html). Pictures 6 and 7 are from a Sept 29, 2009 field trip to Chuxiong, Yunnan (taken by K.L. McNally). Later that day, Dr. Brar received the Yunnan Provincial Government Friendship Award for Foreign Experts at a special provincial banquet held in Kunming in celebration of the 60th anniversary of the founding of the People's Republic of China.

## Ethics Statement

Written informed consent was obtained from the relevant individual(s) for the publication of any potentially identifiable images or data included in this article.

## Author Contributions

All authors listed have made a substantial, direct and intellectual contribution to the work, and approved it for publication.

## Conflict of Interest

The authors declare that the research was conducted in the absence of any commercial or financial relationships that could be construed as a potential conflict of interest.

## Publisher's Note

All claims expressed in this article are solely those of the authors and do not necessarily represent those of their affiliated organizations, or those of the publisher, the editors and the reviewers. Any product that may be evaluated in this article, or claim that may be made by its manufacturer, is not guaranteed or endorsed by the publisher.
